# Cognitive Factors Mediate Placebo Responses in Patients with House Dust Mite Allergy

**DOI:** 10.1371/journal.pone.0079576

**Published:** 2013-11-18

**Authors:** Sabine Vits, Elvir Cesko, Sven Benson, Annika Rueckert, Uwe Hillen, Dirk Schadendorf, Manfred Schedlowski

**Affiliations:** 1 Institute of Medical Psychology and Behavioral Immunobiology, University Hospital Essen, University of Duisburg-Essen, Essen, Germany; 2 Department of Dermatology, Venerology and Allergology, University Hospital Essen, University of Duisburg-Essen, Essen, Germany; University of Granada, Spain

## Abstract

**Background:**

Placebo effects have been reported in type I allergic reactions. However the neuropsychological mechanisms steering placebo responses in allergies are largely unknown. The study analyzed whether and to what extend a conditioned placebo response is affecting type I allergic reactions and whether this response can be reproduced at multiple occasions.

**Methods:**

62 patients with house dust mite allergy were randomly allocated to either a conditioned (n = 25), sham-conditioned (n = 25) or natural history (n = 12) group. During the learning phase (acquisition), patients in the conditioned group received the H_1_-receptor antagonist desloratadine (5mg) (unconditioned stimulus/US) together with a novel tasting gustatory stimulus (conditioned stimulus/CS). Patients in the sham-conditioned control group received the CS together with a placebo pill. After a wash out time of 9 days patients in the conditioned and sham-conditioned group received placebo pills together with the CS during evocation. Allergic responses documented by wheal size after skin prick test and symptom scores after nasal provocation were analyzed at baseline, after last desloratadine treatment and after the 1^st^ and 5^th^ CS re-exposure.

**Results:**

Both conditioned and sham-conditioned patients showed significantly decreased wheal sizes after the 1^st^ CS-evocation and significantly decreased symptom scores after the 1^st^ as well as after the 5^th^ evocation compared to the natural history control group.

**Conclusions:**

These results indicate that placebo responses in type I allergy are not primarily mediated by learning processes, but seemed to be induced by cognitive factors such as patients’ expectation, with these effects not restricted to a single evocation.

## Introduction

Allergic responses of type 1 hypersensitivity, including asthma, are affected by psychological factors such as stress and anxiety [1], [2] and can be modulated by interventions other than conventional drug therapy [3]. The responsiveness of allergic reactions to psychological factors is also reflected by high placebo response rates in clinical studies with allergic patients, in which placebos are routinely employed to test the effectiveness of a drug or a treatment [4]–[6]. During the last decade experimental studies demonstrated that the placebo response is mediated primarily via distinct but interrelated mechanisms [7]. Cognitive factors such as patient expectations of the benefit of a treatment as well as associative learning (conditioning) procedures are steering the placebo response across different diseases and physiological systems [8]–[11]. These psychological mechanisms trigger complex neurobiological phenomena involving the activation of distinct brain areas as well as peripheral physiology, including the release of endogenous substrates [12]–[14].

Based on the bi-directional communication between the central nervous system (CNS) and the peripheral immune system [15], learned placebo effects on immune functions have been demonstrated in rodents as well as in humans [16]–[20]. Typically, in these studies, an immunopharmacological agent (unconditioned stimulus/US) is paired with a gustatory or olfactory stimulus (conditioned stimulus/CS) during the acquisition or learning process. After re-exposure to the CS during evocation, the CS is able to mimic the effects of the immunopharmacological drug formerly used as an US [16]. These learned immunological responses have also been shown to affect allergic responses of type 1 hypersensitivity [21]–[23]. More recently, in an experimental approach with the anti-histamine receptor antagonist desloratadine, an anti-allergic reaction in patients with house dust mite allergy could be demonstrated, reflected by a reduced wheal size in skin prick test and allergic symptom score as well as reduced basophile activation [24].

It remains unclear however, whether the anti-allergic placebo response is primarily induced by cognitive factors such as the expectation of patients towards the benefit of the treatment or by associative learning processes. The knowledge about the underlying neuropsychological mechanisms mediating the anti-allergic placebo responses is essential not only for better controlling the placebo response in clinical trials [11]. It will also form the basis for utilizing the placebo response in the clinical care of allergic patients as a supportive intervention to maximize the pharmacological treatment effects [10], [17], [25].

Thus, in an experimental approach, patients with house dust mite allergy were either exposed to a conditioning protocol or a sham-conditioning protocol to control for effects of expectation, or allocated to a natural history group.

## Methods

### Ethics Statement

The study protocol was approved by the Ethics Committee at the University Hospital Essen. All patients gave written informed consent to take part in the study.

### Patient Recruitment and Inclusion/Exclusion Criteria

Patients with house dust mite allergy were recruited through advertisements and were first screened in a telephone interview. Exclusion criteria were age under 18 years, a specific immunotherapy treatment in the past year, known allergy to desloratadine, allergic asthma, cardiovascular diseases and chronic illnesses. Inclusion criterion for participation was a positive allergic reaction to dermatophagoides pteronyssinus, defined as a wheal size of at least 5 mm in the skin prick test and at least 2 points in the symptom score after nasal provocation [26]. Subjects with known seasonal allergy were included in the study during off season. Subjects resigned, if present, from their usual medication regarding the house dust mite allergy at least two weeks before onset and throughout the study.

In addition patients completed the State-Trait-Anxiety-Inventory (STAI) [27] and the Becks Depression Inventory (BDI) [28] in order to document any group difference in psychological traits variables that might influence the allergic response.

### Study Design

**Figure 1:**
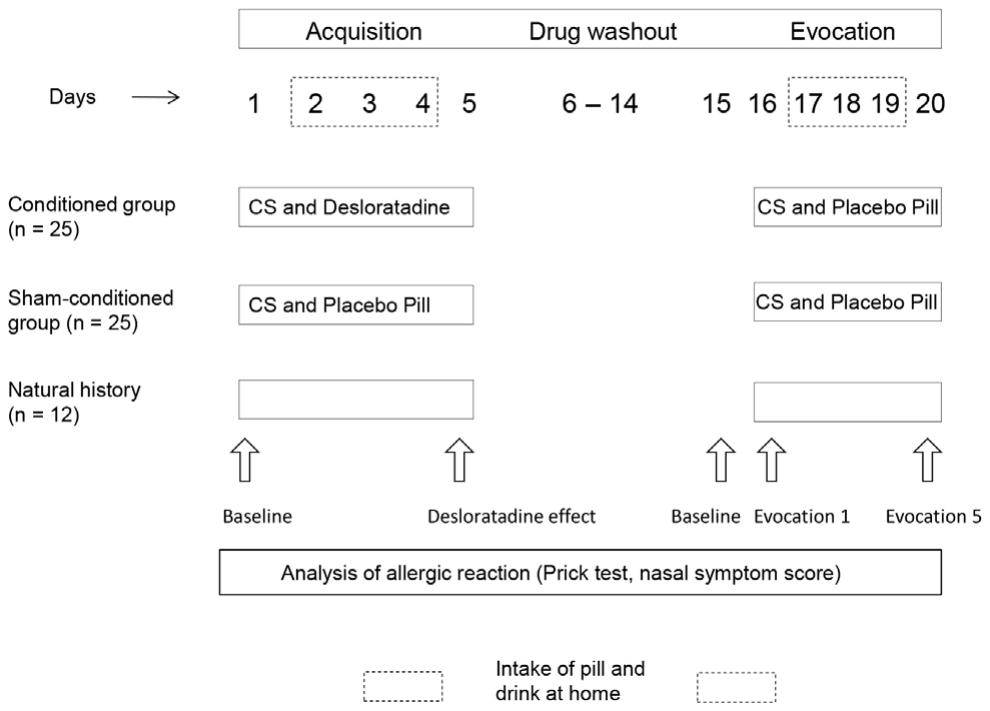
Experimental design. During acquisition, patients in the conditioned group received desloratadine (US) in combination with the CS. During evocation, the drug was replaced by placebo pills. Sham-conditioned patients received the CS together with placebo pills throughout the study. During 3 subsequent days during acquisition (2–4) and evocation (17–19) patients were instructed to intake the pills together with the drink (CS) at home.

Patients were randomly allocated to one of three groups: the conditioned group underwent a conditioning paradigm comprising five days of desloratadine (US) intake together with the gustatory stimulus (CS) in the first phase of the experiment (acquisition phase) (Figure 1). The sham-conditioned group received placebo pills instead of the drug together with the gustatory stimulus during acquisition. During evocation, both groups received placebo pills together with the CS (gustatory stimulus). The natural history group was not exposed to any stimuli. Patients in the conditioned and the sham-conditioned groups were informed that they took part in a study that investigated psychological factors that could influence the effect of the drug desloratadine and that they had 50% chance to either receive the drug or a placebo during the study. Thus, both groups were exposed to identical conditions except that the conditioned group received an active drug in the acquisition phase. Patients in the natural history group were informed that the aim of the study was to analyze the stability of the allergic response over time.

The experiment was performed over a time period of 19 days and included 5 days of acquisition, a drug washout of nine days separating acquisition and evocation phase and five evocation days (Figure 1). For each patient a fixed time for all measurements was set to control for possible intra-individual variance in the allergic reaction due to circadian influences. At baseline, patients first underwent the Skin Prick Test and a nasal provocation test. Afterwards, patients in the conditioned group received a blue colored capsule containing 5 mg of the H_1_ receptor antagonist desloratadine or an identically looking placebo pill in the sham-conditioned group, respectively. Patients were asked to swallow the pill together with 100 ml of a novel tasting green drink (a coloured strawberry milk that was additionally made unfamiliar with lavender oil to form the conditioned stimulus new and salient [18]–[20], [24] that was delivered in a plastic vial. Subsequently, three more vials together with three pills were handed out to the patients. Patients were advised to consume the drink (CS) in parallel with the intake of the pill at the same time of the day for the next three days. This approach was chosen to insure the clinical environment would not become part of the conditioned stimulus, and thus to increase the external validity of the study. On the 5^th^ day patients returned to the lab and were given again a pill and the CS and were then asked to wait for one hour until allergological testing was performed (nasal provocation, skin prick test).

After a drug washout phase of nine days, baseline measures were analyzed on day 15 to control for residual effects of the drug (Fig. 1). On day 16, all patients in the conditioned and the sham-conditioned groups received placebo pills together with the gustatory stimulus (CS). Patients subsequently waited again for one hour until assessment of allergic responses. Three placebo pills and three vials containing the CS were handed out again and patients returned on day 20 for a final assessment of allergic responses after the 5^th^ evocation,. The allergic reaction of natural history patients was documented each time without giving any stimuli to the subjects.

The study was designed as a randomized, double-blind trial. Patients were not informed about their group allocation except patients in the natural history group who did not receive any treatment. The dermatologist performing the skin prick test and the nasal provocation test was blinded with regard to group allocation for all patients.

### Measurement of Allergic Reaction

#### Skin prick test

Skin Prick Tests were performed at the volar site of the forearm with Dermatophagoides pteronyssinus (Der p) extract using ALK Abello lancets. Histamine hydrochloride (10 mg/ml) was used as positive control and NaCl 0.9% as negative control. The results were analyzed after 20 minutes by a dermatologist who was not informed about the patients’ group allocation. Wheal sizes were measured with a stencil.

#### Nasal Provocation Test (NPT) and symptom score

Nasal allergen challenge was performed in accordance to the recommended procedure of nasal provocation tests with allergens used for illness of upper airways by the German Society for Allergological and Immunological Research [26]. Pre challenge, subjects were acclimatized for 30 minutes in the test room and then administered 1 puff of control solution (sodium chloride) in the more consistently nostril, 15 minutes later 2 puffs of allergen were administered. 15 minutes later the allergic response was quantified by using a validated composite symptom score system which comprises the following symptoms: nasal secretion (without = 0, moderate = 1, severe = 2), sneezing (0–2 = 0, 3–5 = 1, >5 = 2), lacrimation/itching of palate/itching of ear (1 point if at least one symptom applies) and conjunctivitis/chemosis/urticaria/coughing/dyspnoe (2 points if at least one symptom applies). The total symptom score was calculated as the sum of the scores for each symptom (maximum score: 7).

### Statistical Analysis

Changes in wheal size were analyzed using a repeated measure ANOVA for the acquisition phase and the evocation phase, respectively. In case of a significant interaction effect, post-hoc t-tests for pairwise comparisons were performed for each time point during the acquisition and evocation phase. Non-parametric testing was performed for the symptom score due to ordinal scaling of this variable. Kruskal-Wallis test was applied for each time point during both phases to detect any differences between groups. If significant results were obtained, a Mann-Whitney U-Test was performed for pairwise comparisons. All data are expressed as mean ±SEM. SPSS 20.0 was used for all analyses. Statistical significance was defined as a p- value <0.05.

## Results

### Patient Characteristics

63 patients (37 female, 25 male) were recruited for the study and randomly allocated to one of the three groups: conditioning, sham-conditioning or natural history. 62 patients were included in the final analysis. One patient developed a cold during the study making nasal provocation testing impossible. Patient groups did not statistically differ with regard to sex, age, trait anxiety or depressive symptoms (Table 1).

**Table 1:** Sociodemographic and psychological characteristics.

	Conditioned group (n = 25)	Sham-conditioned group (n = 25)	Natural history group (n = 12)	Statistics for group comparison*	Total (N = 62)
Sex					
female n (%)	15 (60)	17 (68)	5 (41,7)	?^2^ = 2.3,p = 0.31	37 (59,7)
male n (%)	10 (40)	8 (32)	7 (58,3)		25 (40,3)
Age					
mean (SD)	31.7 (10.6)	27.9 (7)	32.6 (6.1)	F = 1.7,p = 0.18	30.3 (8.6)
STAI X2					
mean (SD)	34.3 (7.6)	36.7 (8.6)	34.4 (6.1)	F = 0.7, p = 0.52	35.3 (7.7)
BDI					
mean (SD)	4 (3.3)	4.6 (4.7)	5.3 (3.2)	F = 0.4, p = 0.65	4.5 (3.9)

BDI = Becks Depression Inventory; STAI X2 = Trait anxiety form of the State-trait anxiety inventory.

* Results of Chi^2^ Test or univariate ANOVA.

### Wheal Size

**Figure 2:**
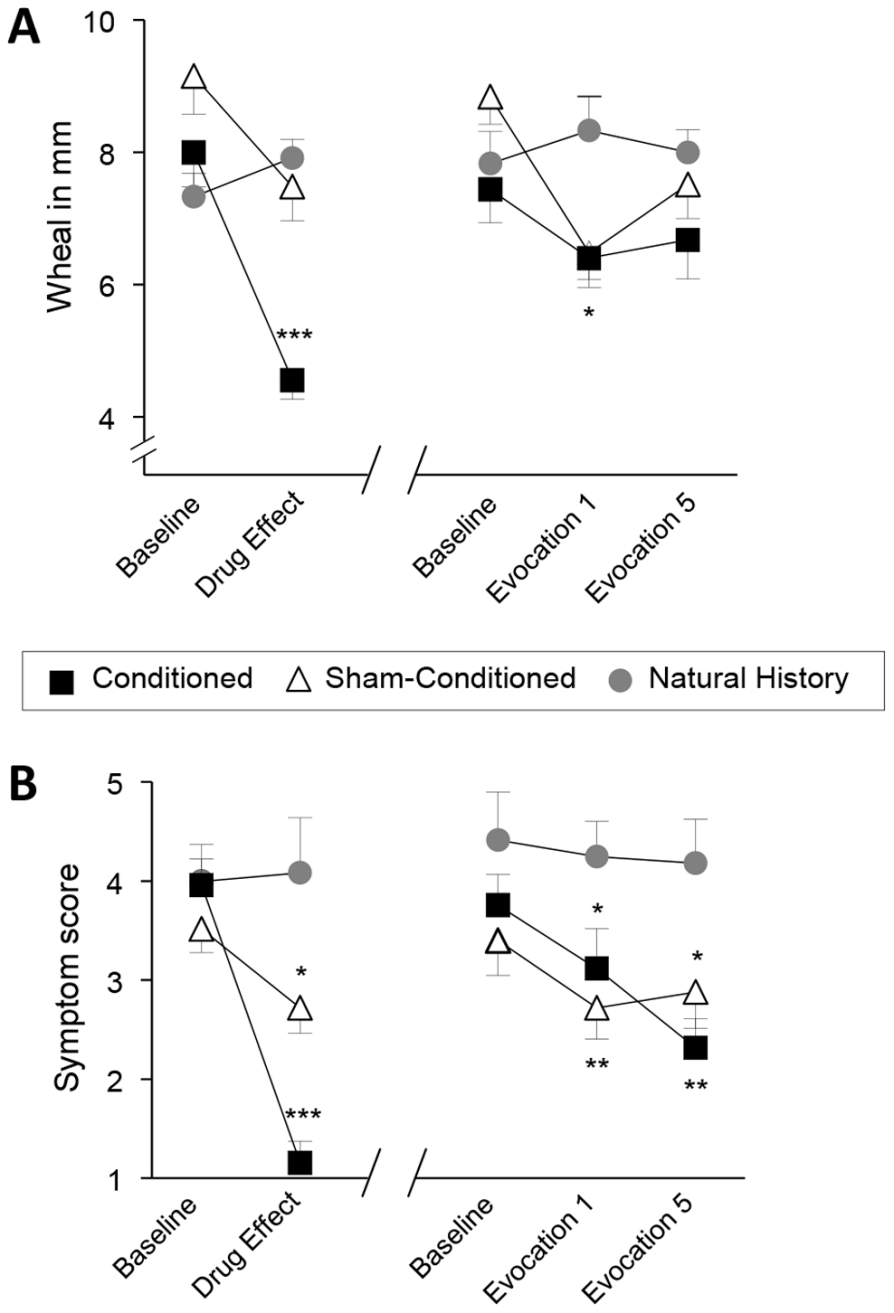
Conditioned and sham-conditioned patients show reduced allergic reactions during evocation. **A** After skin prick test wheal size (mm) was analyzed before and after acquisition as well as before and after the 1^st^ and 5^th^ evocation to the CS in patients in the conditioned, sham-conditioned as well as patients in the natural history group. **B** Symptom scores after nasal provocation were analyzed before and after acquisition as well as before and after the 1^st^ and 5^th^ evocation to the CS in patients in the conditioned, sham-conditioned as well as patients in natural history group. Data are presented as mean ±SEM. *p<0.05 **p<0.01 ***p<0.001, comparison against natural history group.

After acquisition, mean wheal sizes significantly differed between the three groups (ANOVA interaction effect F_(2,59)_ = 12.6, p<0.001) with significantly reduced wheal sizes in patients receiving desloratadine (conditioned group) in comparison to the natural history group (t_(35)_ = −7.1, p<0.001) or sham-conditioned group of patients (t_(35)_ = 4.9, p<0.001) (Fig. 2A), respectively. In contrast, wheal sizes in the sham-conditioned group were not significantly reduced compared to the natural history group (t_(48)_ = −0.7, p = 0.46).

After nine days of drug washout, conditioned and natural history patients did not differ in wheal sizes (t_(35)_ = −0.5, p = 0.63), indicating no residual drug effects. During evocation, analyses of the allergic response indicated significant differences in wheal size between the three groups (ANOVA interaction effect, F_(4,118)_ = 2.51, p = 0.046) with significantly decreased wheal sizes in conditioned patients compared to patients in the natural history group after the 1^st^ evocation (t_(35)_ = −2.7, p = 0.01) (Fig. 2A). Interestingly, a comparable attenuation of the allergic response was also observed in the sham-conditioned group (t_(35)_ = −2.7, p = 0.01 vs. natural history), and wheal sizes did not significantly differ between conditioned and sham-conditioned group (t_(48)_ = 0.1, p = 0.89). In contrast, after the 5^th^ evocation no group differences were found (conditioned vs. natural history group t_(35)_ = −1.9, p = 0.06; sham-conditioned vs. natural history group t_(35)_ = −0.8, p = 0.45; conditioned vs. sham-conditioned t_(48)_ = 1.1, p = 0.29).

### Symptom Score

Analogous to wheal size analyses, after receiving either desloratadine (conditioned group*)*, or placebo pills (sham-conditioned group*)* for five days, significant differences between the three groups were observed (χ^2^_(2)_ = 24.3, p<0.001) (Fig. 2B). Interestingly, in addition to the expected drug effect in the conditioned group in which patients received the drug (z_(35)_ = −4.07, p<0.001 compared to natural history; z_(48)_ = −3.9, p<0.001 compared to sham-conditioned patients), a significant reduction in symptom scores was also observed in sham-conditioned patients when compared to the natural history group (z_(35)_ = −1.97, p = 0.048).

Again, no residual drug effect was observed after nine days of drug washout, reflected by comparable symptom scores in all study groups (χ^2^_(2)_ = 2.94, p = 0.23) at baseline measures before the evocation phase. However, groups significantly differed in symptom scores after the 1^st^ (χ^2^_(2)_ = 8.13, p = 0.02) as well as after the 5^th^ fifth (χ^2^_(2)_ = 8.85, p = 0.01) evocation (Fig. 2B). In response to placebo treatment, symptom scores at both evocations were significantly decreased in conditioned patients compared to patients in the natural history group (1^st^ evocation, z_(35)_ = −2.11, p = 0.04; 5^th^ evocation z_(35)_ = −2.95, p = 0.003). However, the same response was observed in the sham-conditioned group (1^st^ evocation z_(35)_ = −2.9, p = 0.004; 5^th^ evocation z_(35)_ = −2.2, p = 0.03), with no group differences between conditioned and sham-conditioned patients at both evocations (1^st^ evocation z_(48)_ = −0.7, p = 0.46; 5^th^ evocation z_(48)_ = −1.0, p = 0.31).

## Discussion

In this study we employed a conditioning protocol to investigate learned placebo responses in patients with house dust mite allergy. After five days of desloratadine intake patients in the conditioned group showed the expected reduction of wheals sizes and symptom scores. In addition, a significant reduction in symptom scores was also observed in the sham-conditioned group. During the evocation phase, patients in the conditioned group showed a significantly reduced wheal size after the 1^st^ but not after the 5^th^ evocation. In contrast, symptom scores were significantly reduced after the 1^st^ as well as after the 5^th^ evocation trial in comparison to the natural history group. Interestingly, sham-conditioned patients also showed significant reductions in wheal sizes and symptom scores and did not statistically differ from patients in the conditioned group, indicating a placebo response primarily induced by expectation rather than via associative learning processes.

Cognitive factors such as expectation of patients towards the benefit of a forthcoming treatment and associative learning processes have been demonstrated as crucial neuropsychological factors steering the placebo response [8]–[10]. However, which of these two factors is mediating the placebo response in various clinical conditions is largely unknown. Expectation is mediating placebo effects in many clinical conditions such as pain or Parkinson [29]–[31] and expectation induced placebo analgesia can be maximized through prior learning experience [32]. However, autonomous functions such as hormone release or peripheral immune functions are believed to be primarily affected by associative learning processes and not by mere cognitive factors such as expectation [33], [34]. In allergic diseases, data on the effects of expectation-induced placebo responses on objective and clinical relevant measures are somewhat controversial. A pronounced placebo response was observed in bronchial hyper-reactivity in asthma patients [35]. More recently, expectation-induced placebo effects improved the subjective well-being in asthma patients, however did not affect the forced expiratory volume analyzed by spirometry [36]. In patients with house dust mite allergy, the subjective total symptom rating as well as the skin prick test was significantly reduced by placebo responses induced by a learning protocol as well as via expectation; however, *ex vivo* basophile activation was significantly suppressed only in patients undergoing the learning protocol but not by mere expectation [24]. In our study, the mere expectation of receiving an active drug with a probability of 50% induced a placebo response in wheal size and symptom score during the evocations in the sham-conditioned group that was as pronounced as the learned placebo response in the conditioned group. These results confirm earlier observations of an expectation-induced placebo response in objective and clinical relevant measures as a component of allergy treatment [4], [5].

The sustainability and reproducibility of placebo responses in different clinical conditions is still an open question [9], [11], [17]. We could recently demonstrate that a learned placebo response on the inhibition of cytokine release in healthy subjects can be reproduced by mere re-exposure to the taste stimulus used as a CS [18]. In this study, a significant inhibition in symptom score was observed after the 1^st^ but also after the 5^th^ unreinforced re-exposure to the CS in the conditioned but also sham conditioned patients, indicating that the placebo response in house dust mite allergy is not restricted to a single event. However, how long-lasting this placebo response on the allergic reaction is has to be analyzed in further studies.

When designing this study we intended to test the effectiveness of the learning protocol under partial “natural conditions”. Thus, during acquisition and evocation phases patients were asked to take the drink (CS) as well as the drug or placebo respectively at home. On the one hand this design increased the external validity of our data, on the other hand, this might have also limited the effect size in the conditioned group. The hospital environment and medical atmosphere together with the doctor who delivers the pill and the drink might have been important contextual cues further augmenting the conditioned effect [37]. In addition, although all patients confirmed drug and CS intake at home, the lack of control of patients’ compliance is certainly limiting the interpretation of these data.

In this study, we induced the expectation of a 50% probability to receive an active drug during each pill intake. Since experimental and clinical studies show that the likelihood of being in a treatment group increases the size of placebo responses [30], [38]–[40] it remains to be investigated whether the employment of a deceptive paradigm that induces a 100% expectation of receiving an active drug although actually a placebo is given [32], [35], [41], [42] will induce a more pronounced placebo response in allergic reactions. Also, an interesting approach with regard to the applicability of placebos in clinical routine for the benefit of the patient, is the administration of open-label placebo [43], [44], which remains to be tested for its effectiveness in allergy.

In summary, these data demonstrate placebo responses on wheal sizes after skin prick test and symptom scores in patients with house dust mite allergy which are induced by behavioral conditioning as well as cognitive expectation. These data might have implications for clinical trials in allergic patients and might provide a basis for systematically employing placebo responses in combination with pharmacological treatment with the aim to maximize treatment outcome for the patient benefit.
